# Molecular Insights into Oil–Water Interfacial Behavior of Gemini Sulfonate Surfactants: The Critical Role of Spacer Group

**DOI:** 10.3390/polym18212665

**Published:** 2026-10-28

**Authors:** Wen Ren, Hao Lu, Jinyuan Zhang, Xingchun Li, Linglan Zhang, Chunmao Chen, Qinghong Wang, Shuixiang Xie, Ling Lin

**Affiliations:** 1 State Key Laboratory of Heavy Oil Processing, College of Chemical Engineering and Environment, China University of Petroleum-Beijing, Beijing 102200, China; 2 State Key Laboratory of Petroleum Pollution Control, CNPC Research Institute of Safety and Environment Technology, Beijing 102206, China; 3 School of Chemistry and Chemical Engineering, Southwest Petroleum University, Chengdu 610500, China

**Keywords:** gemini sulfonate surfactant, spacer group, molecular dynamics, interface

## Abstract

Gemini surfactants are extensively employed in enhanced oil recovery (EOR) owing to their low critical micelle concentration (CMC), excellent interfacial activity, and strong self–assembly capability. However, molecular–level understanding of how spacer chemistry regulates the interfacial behavior of sulfonate–containing gemini surfactants remains limited. All–atom molecular dynamics simulations were employed to investigate four gemini sulfonate surfactants with distinct spacers (carbon–chain spacer (CA), ester containing spacer (EG), benzene–ring spacer (BR), and nitrogen–containing (NA)) at the n–decane/water interface. Based on its superior interfacial performance, the EG system was further selected to evaluate the effects of polyacrylamide (PAM) and partially hydrolyzed polyacrylamide (HPAM). Increasing surfactant concentration markedly altered interfacial assembly, and at 2.33 μmol/m^2^, pronounced interfacial curvature indicated a tendency toward microemulsion-like structures. Spacer chemistry strongly regulated interfacial thickness, hydration, and molecular organization. The rigid aromatic spacer of BR broadened the interfacial region through its strong oil–phase affinity, whereas the ester–containing EG spacer enhanced hydration and hydrogen–bond formation and promoted favorable molecular packing, resulting in the best overall interfacial performance. Further simulations showed that PAM accumulated near the EG interfacial layer and strongly interacted with EG, leading to a pronounced reduction in interfacial tension. In contrast, hydrolysis strengthened HPAM–water interactions and promoted its distribution in the bulk aqueous phase, thereby limiting its influence on the interface. This study provides molecular insights into how different spacer groups regulate interfacial behavior, offering theoretical guidance for the design and formulation of gemini surfactant systems for EOR.

## 1. Introduction

Presently, the global demand for energy, notably petroleum, is undergoing substantial growth. Following primary and secondary extraction, large quantities of crude oil remain unrecovered [1,2,3,4,5], and it is becoming increasingly difficult to recover oil as the recovery rate increases. To enhance oil recovery (EOR), tertiary oil recovery technology has become essential. This technology utilizes chemical additives to modify interfacial interactions among oil, gas, water, and rock formations, potentially increasing crude oil recovery rates to 80–85%. With the continuous development of EOR technologies, surfactants with distinctive molecular structures and performance characteristics have become important research targets, offering broad prospects for the recovery of residual oil [6,7]. Among these surfactants, gemini surfactants have received increasing attention due to their unique molecular architecture and pronounced self–assembly behavior. As amphiphilic molecules, gemini surfactants comprise two hydrophilic head groups, two hydrophobic tails, and a connecting spacer that links two conventional single–chain surfactant molecules [8,9,10]. The spacer chemically links the two hydrophilic headgroups, reducing electrostatic repulsion between them and facilitating dense molecular packing [11,12,13]. It has been demonstrated that the structural configuration, length, and functional groups of the spacer can have a profound effect on the performance of gemini surfactants [14,15,16]. Compared to conventional single–chain surfactants, gemini surfactants exhibit superior characteristics: lower critical micelle concentration (CMC), enhanced surface activity, improved solubilization capacity, and superior wettability [17,18,19,20,21,22]. These advantages have established gemini surfactants as valuable agents for EOR applications [23,24,25].

In recent years, increasing research efforts have been devoted to tailoring the molecular architectures of gemini surfactants to optimize their interfacial and physicochemical properties relevant to oil displacement [26,27,28]. Yan et al. [29] designed anionic gemini surfactants with different hydrophilic headgroups and showed that sulfonate headgroups produced greater reductions in interfacial tension because of their greater hydration. Du et al. [11] synthesized seven sulfonate gemini surfactants with varying hydrophobic chain and spacer lengths, demonstrating that spacer length exerted a greater influence on the phase transition temperature than alkyl chain length. Gu et al. [30] developed sulfate gemini surfactants with rigid or flexible spacers and found that surfactants with rigid spacers exhibited greater thermal stability and lower surface tension at the critical micelle concentration (γ_CMC_), despite having higher CMC values. Morita et al. [31] further showed that cyclic spacers slowed interfacial adsorption owing to their conformational rigidity. Similarly, Cao et al. [16] demonstrated that spacer chemistry regulated adsorption kinetics, interfacial organization, and the resulting physicochemical properties. Lu et al. [32] reported that spacers containing sulfate groups markedly enhanced surface activity. Yarahmadi et al. [33] further showed that longer alkyl tails lowered the CMC, whereas longer spacers increased the CMC, led to looser molecular packing at the interface, and improved salt tolerance. The longer spacers increase the separation between charged headgroups, thereby reducing electrostatic repulsion, while their greater conformational flexibility allows the headgroups to adopt more favorable orientations in the presence of counterions, ultimately improving salt tolerance under high–salinity conditions. Collectively, these studies establish the critical roles of the hydrophilic headgroup, hydrophobic tail, and spacer in governing the interfacial and physicochemical properties of gemini surfactants.

Advances in computational methods have enabled molecular and mesoscale simulations to elucidate how structural variations in gemini surfactants affect their interfacial and self–assembly behavior. Han et al. [34] used dissipative particle dynamics (DPD) simulations to show that cationic gemini surfactants with shorter spacers exhibited greater interfacial activity. Ma et al. [35] found that aromatic groups promoted microemulsion formation by regulating droplet coalescence through π–π interactions and steric effects. Wen et al. [36] demonstrated that the hydrophobic chain and spacer lengths of alkylbenzene sulfonate gemini surfactants jointly regulated their interfacial behavior in dodecane–water systems, thereby affecting nanoscale mixing, the CMC, and transitions from lamellar structures to micelles. Santos–Santos et al. [37] showed that the angular configuration of X–shaped gemini surfactants governed the stability of oil–surfactant aggregates and the efficiency with which these aggregates encapsulated monomeric oil, with the stretched configuration consistently exhibiting higher encapsulation efficiency. Man et al. [38] reported that gemini surfactants containing EO groups and both anionic and nonionic moieties exhibited high surface activity, with their self-assembled structures transitioning from vesicles to micelles as the hydrophobic chain length increased. Bai et al. [39,40] further demonstrated that spacer length regulated the interfacial co-assembly and hydration of amine gemini surfactants with sodium oleate or octanol, thereby affecting water mobility, foam drainage, and foam stability. In chemical enhanced oil recovery, surfactants are often combined with polyacrylamide (PAM) or partially hydrolyzed polyacrylamide (HPAM) [41]. Previous studies have shown that polymer–surfactant interactions can modify interfacial adsorption and molecular organization, thereby affecting oil–water interfacial properties [42,43].

However, despite extensive investigations into gemini surfactants with diverse headgroups, tail–chain lengths, and spacer architectures, the molecular–level mechanisms by which the chemical characteristics of spacer groups, such as polarity, hydrophobicity, rigidity, and flexibility, govern interfacial behavior remain insufficiently understood. Therefore, four gemini sulfonate surfactants with distinct spacer architectures were selected as representative model systems to investigate the role of spacer chemistry in regulating interfacial behavior. The selected surfactants contain different types of spacer groups, including a flexible hydrophobic carbon–chain spacer (CA), a polar ester–containing spacer (EG), a nitrogen–containing spacer (NA), and a rigid aromatic spacer (BR). All-atom molecular dynamics simulations were employed to investigate how these spacer structures affect interfacial adsorption, oil–water interactions, hydration behavior, and molecular packing. Furthermore, based on the comparative evaluation of the four surfactant systems, a representative system with favorable interfacial performance was further investigated in the presence of PAM and HPAM with a hydrolysis degree of 20% to examine the effects of polymer addition on interfacial organization and interfacial properties. Overall, this study aims to provide molecular–level insights into the relationship between spacer chemistry and interfacial behavior and to further elucidate the influence of polymer addition on a representative gemini surfactant system.

## 2. Models and Simulation Details

### 2.1. Model Construction

**Figure 1:**
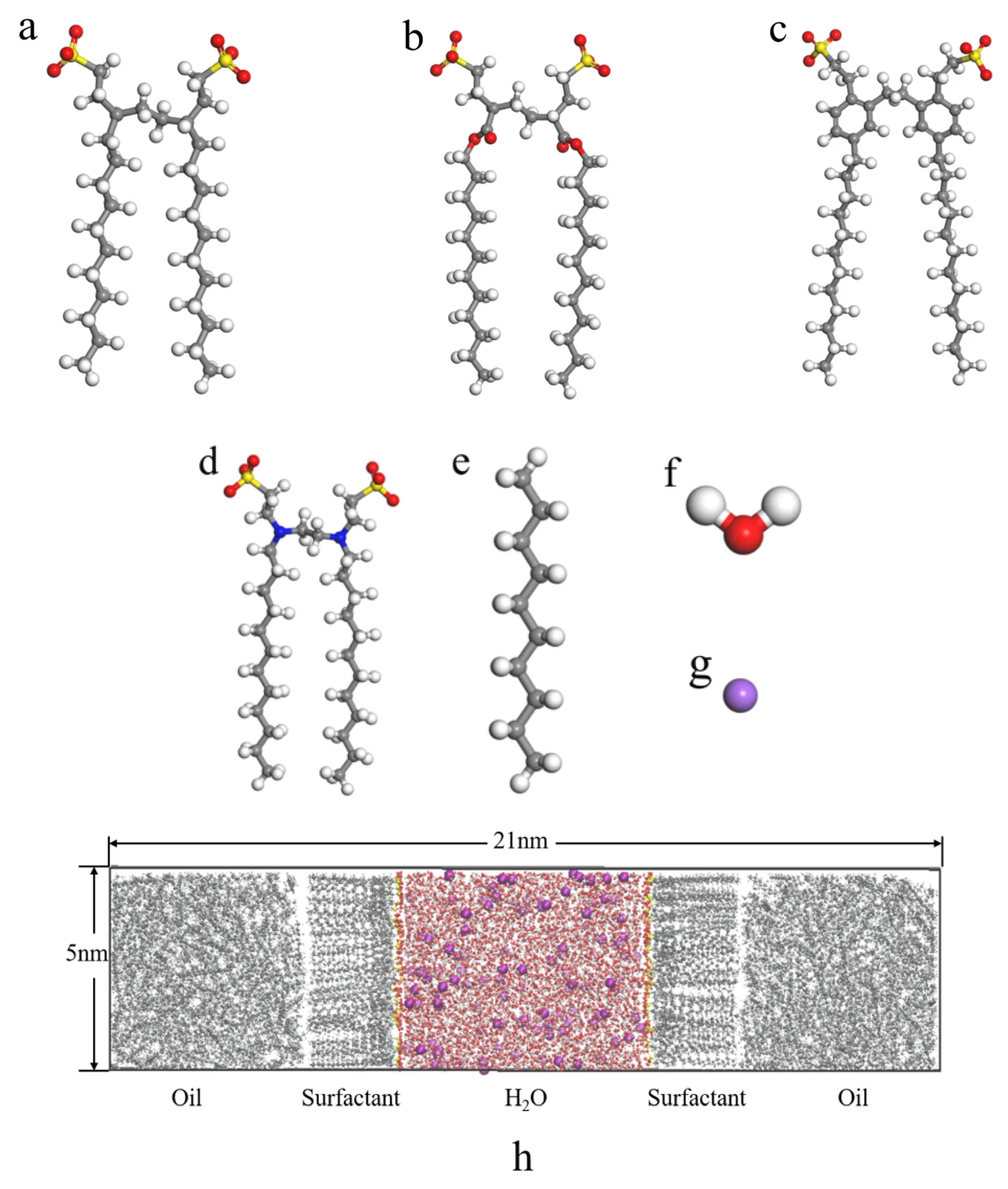

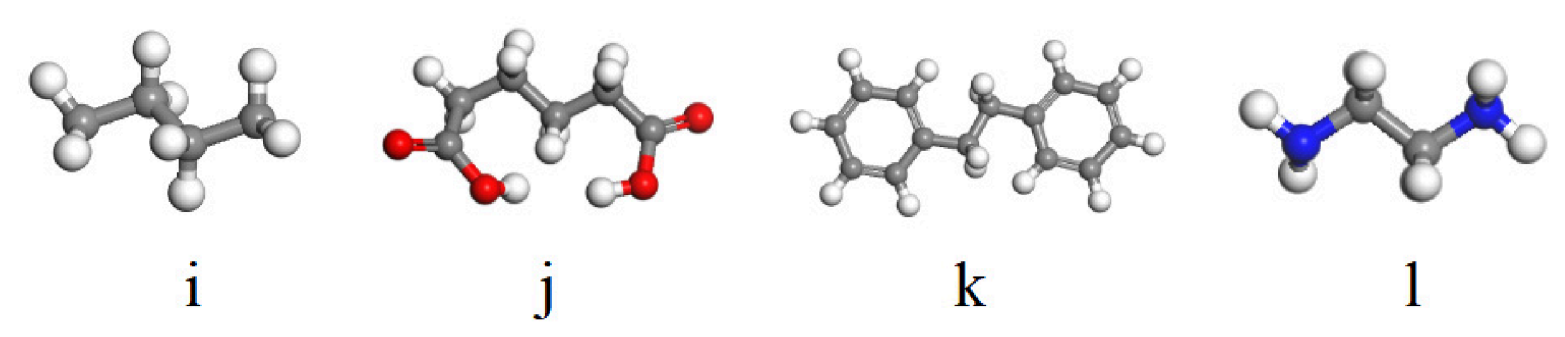
(**a**–**g**) Structures of surfactants CA, EG, BR, NA, n-decane, water, and sodium ion. (**h**) Initial model of the system. (**i**–**l**) Structures of the spacer groups in CA, EG, BR, and NA, respectively. The carbon atom is colored grey, the hydrogen atom is colored white, the oxygen atom is colored red, the sulfur atom is colored yellow, the nitrogen atom is colored blue, and the sodium ion is colored purple.

This work adopted a sandwich–type model, whose simulation box measured 5 nm × 5 nm × 21 nm. The system consisted of two n–decane phases separated by an aqueous layer, with surfactants initially placed at both oil–water interfaces [44,45,46]. Each oil phase contained 350 n-decane molecules, while the water phase included 5000 molecules modeled using the OPC water model, which exhibits high accuracy in reproducing bulk water properties [47,48]. For each surfactant system, five different concentrations were selected, and the interfacial concentrations were set as 1.00, 1.33, 1.67, 2.00 and 2.33 μmol/m^2^ (i.e., 15, 20, 25, 30, and 35 surfactant molecules per interface). Hydrophilic headgroups were oriented toward the aqueous region and hydrophobic tails toward the oil region in the initial molecular arrangement, which accelerated the equilibration process. Sodium ions were subsequently introduced to each system for charge balance. The whole set of molecules and ions was packed into the simulation box via the Packmol package [49]. To further investigate the effects of polymer addition, the EG system at an interfacial concentration of 1.67 μmol/m^2^ was selected for the polymer–containing simulations. The EG system was supplemented with either two PAM chains or two HPAM chains. Both PAM and HPAM had a polymerization degree of 20 [50]. For HPAM, a hydrolysis degree of 20% was adopted, corresponding to four acrylate units randomly distributed along the polymer backbone [51,52]. The other components were kept unchanged, and an appropriate number of Na^+^ counterions was added to ensure system electroneutrality. In total, 23 simulation systems, including a pure oil–water interface system as the reference, were established, with the detailed parameters summarized in Table 1. Molecular models were created with the Materials Studio software (2017 version). Figure 1 presents both the molecular structures and the initial configurations for the simulated systems.

### 2.2. Molecular Dynamics

All MD simulations were run via the LAMMPS package [53]. Bonded and non-bonded interactions of n-decane, surfactants, PAM, and HPAM were modeled with the OPLS-AA force field [54,55,56], with the interaction potential defined in Equation (1):

(1) $$E_{\mathrm{ab}}=\sum_{i}^{a}\;\sum_{j}^{b}\;\left[\frac{q_{i}q_{j}e^{2}}{r_{\mathrm{ij}}}+4\varepsilon_{\mathrm{ij}}\left(\frac{\sigma_{\mathrm{ij}}^{12}}{r_{\mathrm{ij}}^{12}}-\frac{\sigma_{\mathrm{ij}}^{6}}{r_{\mathrm{ij}}^{6}}\right)\right]$$

where *r_ij_* is the distance between atoms *i* and *j*, *ε_ij_* and *σ_ij_* are the Lennard–Jones radius and well depths of the atomic pair (*i*, *j*), *q_i_* and *q_j_* indicate the charges of atoms *i* and *j*, respectively, and e is the elementary charge. For interactions between different atom types, their van der Waals potential parameters are calculated using the geometric mean mixing rules, as described by Equation (2):

(2) $$\epsilon_{\mathrm{ij}}=\sqrt{\epsilon_{i}\epsilon_{j}},\sigma_{\mathrm{ij}}=\sqrt{\sigma_{i}\sigma_{j}}$$

The 1.2 × CM5 method was employed to derive atomic charges. This approach, in combination with the OPLS–AA force field, has been reported to accurately capture the oil–water interfacial properties [57,58,59]. Temperature and pressure for all simulations were maintained at 298.15 K and 1 atm using the Nosé–Hoover thermostat and barostat, with relaxation times of 0.1 ps and 1.0 ps, respectively [60]. For the MD simulations, a 1 fs time step was used, with configurations written every 10 ps for later analysis. A 10 Å cutoff was applied to short–range forces, while long–range electrostatics were handled via the particle–particle particle–mesh (PPPM) method to an accuracy of 10^−5^ [61]. The SHAKE algorithm was applied to fix bond lengths and angles of water molecules. For each system, an initial geometry optimization using the steepest descent algorithm was performed, then 500 ps equilibration runs were carried out in both NVT and NPT ensembles. This method enabled stable membrane assembly at the oil/water interface while eliminating vacuum and allowing the system to reach a reasonable density. Finally, data were extracted from a 5 ns production run under NVT. All MD trajectories were visualized using the VMD 2.0 software package [62].

## 3. Results and Discussion

### 3.1. Validation of Force Field and Model

**Figure 2:**
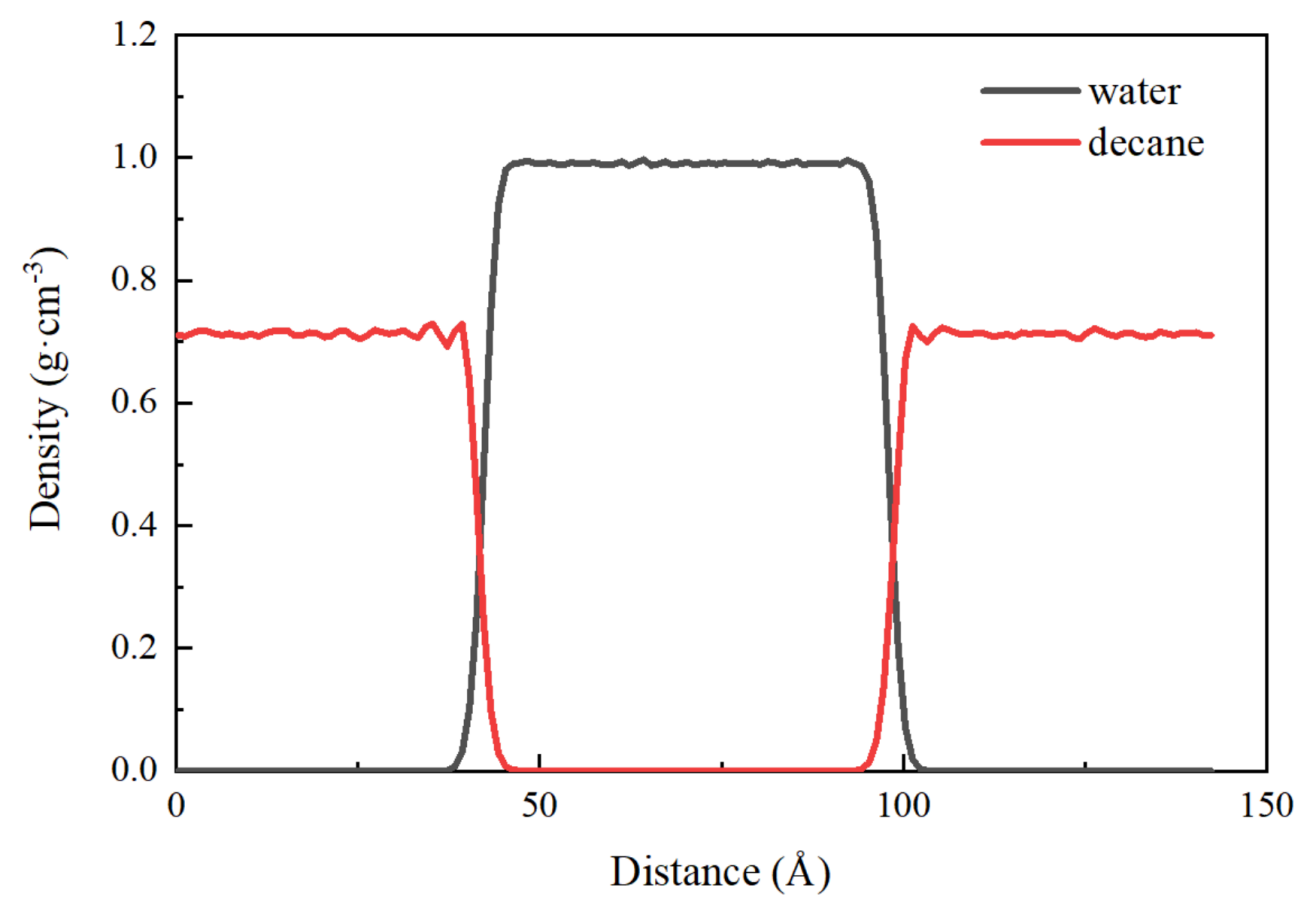
Density distribution of the water/n–decane system. The black line represents the water phase, and the red line represents the oil phase.

The OPLS–AA force field was validated for the n–decane/OPC water system. The validation was performed based on the simulated density profiles and interfacial tension of the pure oil–water interface. The z–axis density profiles for the water/n-decane system are presented in Figure 2. Water exhibits a density of approximately 0.991 g/cm^3^, while n–decane shows about 0.713 g/cm^3^. These values agree well with the reported experimental data (0.998 and 0.725 g/cm^3^) at 298.15 K and 1 atm [63,64]. Interfacial tension is a key parameter for characterizing oil–water interfaces. This quantity is commonly employed to check the accuracy of different force fields in describing such systems [65,66,67]. In MD simulations, interfacial tension is evaluated from the normal and tangential pressure (Pn and Pt) components at the interface, as expressed by Equation (3):

(3) $$\gamma =\frac{1}{2}L_{z}\left[P_{\mathrm{zz}}-\frac{1}{2}\left(P_{\mathrm{xx}}+P_{\mathrm{yy}}\right)\right]$$

In this expression, P_zz_ corresponds to the pressure component perpendicular to the interface, whereas P_xx_ and P_yy_ are the pressure components parallel to the interface direction. L_z_ represents the box dimension along the z axis. The simulations yield an interfacial tension of 54.04 mN/m for the pure water/n-decane system, which is in close agreement with the experimental value of 52.50 mN/m at 298.15 K and 1 atm [68]. Therefore, based on the agreement in bulk density and interfacial tension, the OPLS–AA force field and the present model are considered suitable for describing microscopic oil–water interfacial interactions.

### 3.2. Interface Morphology and Density Distribution

**Figure 3:**
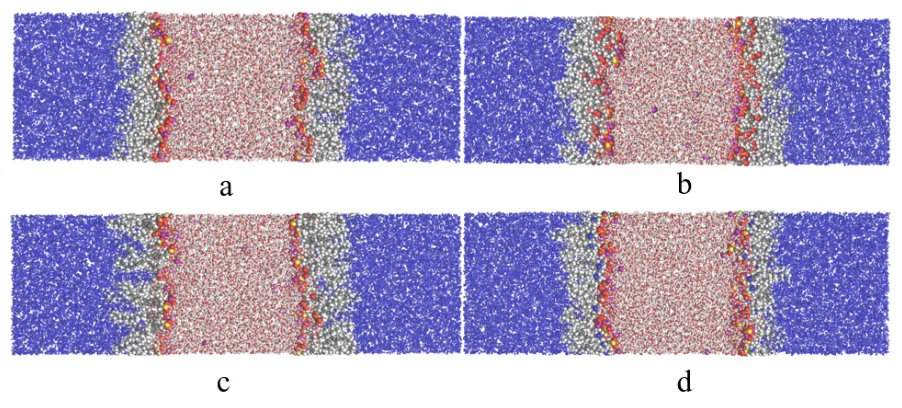
Final snapshots of the four systems at an interfacial surfactant concentration of 2.00 μmol/m^2^: (**a**) CA, (**b**) EG, (**c**) BR, and (**d**) NA. The oil phase is shown in blue and the water phase in light red. In the surfactant molecules, carbon, hydrogen, oxygen, sulfur, and nitrogen atoms are shown in grey, white, red, yellow, and blue, respectively, while sodium ions are shown in purple.

**Figure 4:**
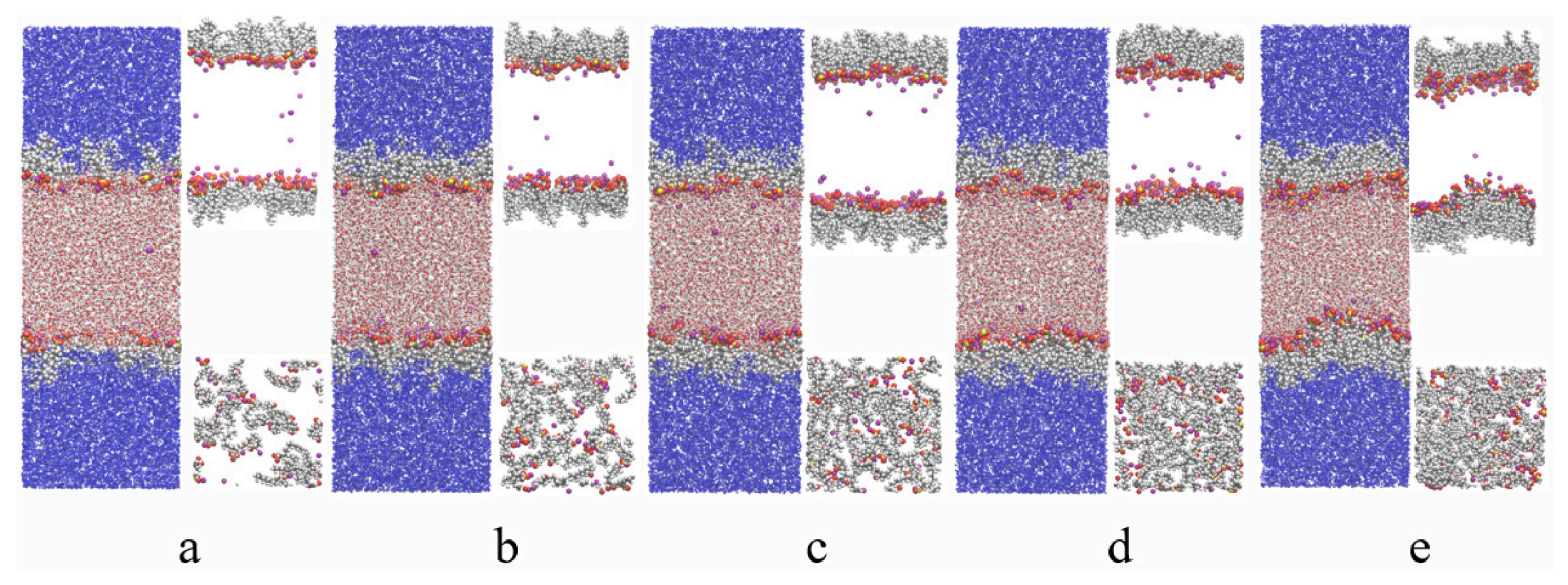
The last frame snapshot after the simulation of different concentrations of CA system, as well as the front view and top view after removing the water phase and oil phase. (**a**) 1.00, (**b**) 1.33, (**c**) 1.67, (**d**) 2.00, (**e**) 2.33.

To study the interfacial morphology of the four surfactant systems, five sets of MD simulations at varying concentrations were performed for CA, EG, BR, and NA. The density distribution and interface aggregation morphology of each system were compared by changing the interfacial surfactant concentration. Figure 3 displays that at an interfacial surfactant concentration of 2.00 μmol/m^2^, upon simulation completion, all four surfactants formed a well–distributed film at the oil–water interface. The hydrophilic heads resided near the water phase, while the hydrophobic tails pointed toward the oil phase, creating an adsorption layer between the two phases. Figure 4 reveals that the majority of sodium ions aggregate in the interfacial layer, driven by electrostatic attraction to the negatively charged sulfonate groups, while the remainder stay free in the water phase, maintaining a dynamic equilibrium with those at the interface. For surfactant systems with different concentrations, it can be seen from Figure 4 that when the surfactant interfacial concentration is 1.00 μmol/m^2^, a limited number of surfactant molecules are randomly distributed at the interface. At this time, the surfactant is loosely arranged, and there is a large gap at the interface. Some water molecules are in direct contact with n–decane molecules, but the surfactant layer remains relatively flat. With increasing surfactant concentration, the interfacial surfactant layer becomes progressively denser. When the surfactant interfacial concentration is 2.33 μmol/m^2^, the surfactant layer exhibits bending behavior. With increasing surfactant concentration, the molecular arrangement at the oil–water interface becomes progressively denser. Once the interfacial concentration exceeds the maximum packing capacity of the planar adsorption layer, the planar arrangement can no longer be maintained and the interfacial film begins to bend. With further surfactant accumulation, this deformation becomes more pronounced, and the aggregation morphology gradually evolves from a planar interfacial layer into microemulsion–like structures.

**Figure 5:**
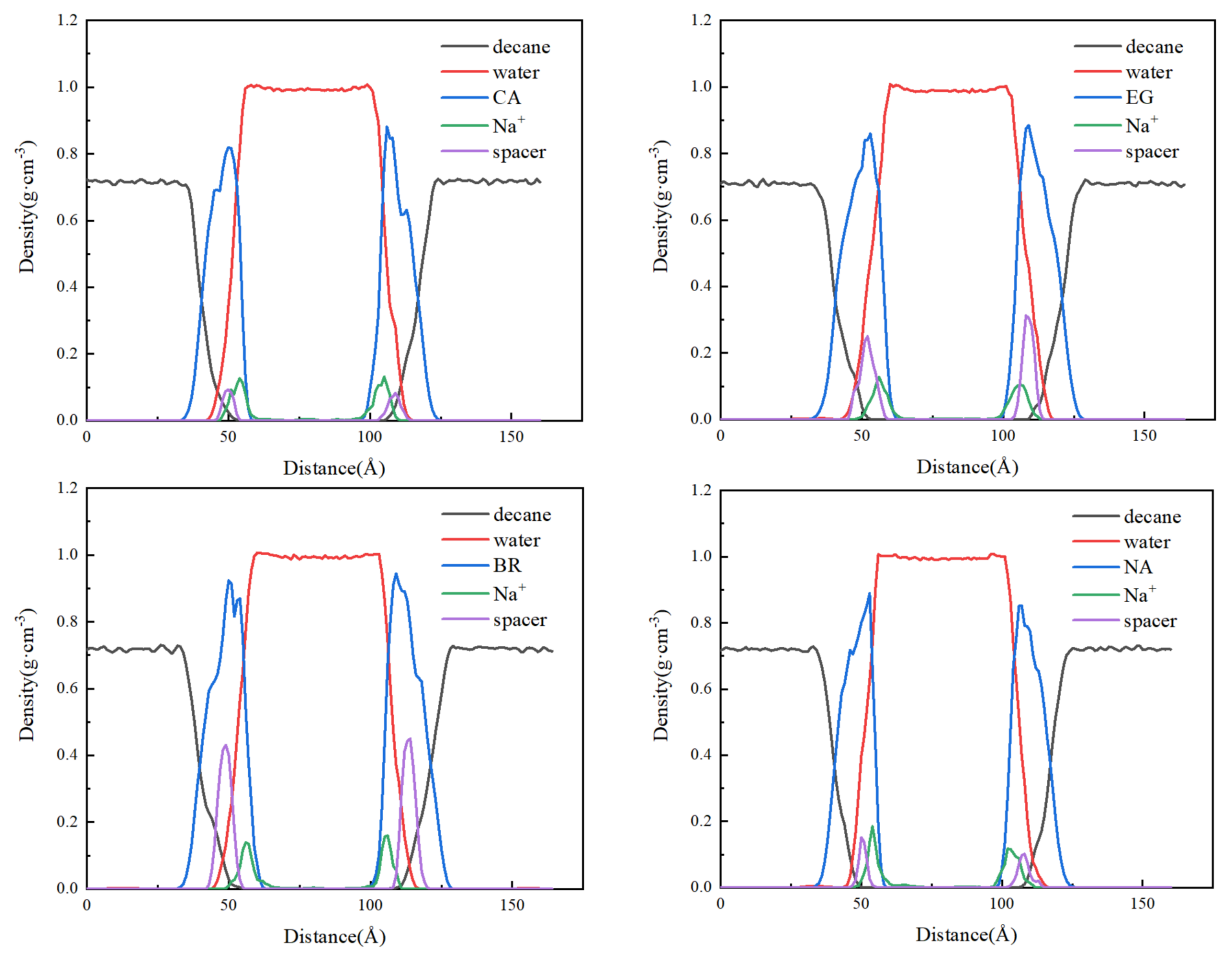
When the interfacial surfactant concentration is 2.00 μmol/m^2^, the density distribution of each component along the z–axis.

In order to study the distribution of each substance in the four surfactant systems, an analysis was performed on the density distribution of surfactant, n–decane, water, sodium ion and spacer group along the z–axis direction in the four simulated systems. As shown in Figure 5, after 5 ns of MD simulation, the bulk phase density of water and n–decane tends to be gentle and stable, while the surfactant molecules and sodium ions show symmetrical double peaks at the two interfaces, indicating that both surfactants and sodium ions are mainly distributed at the interface. It can also be seen from Figure 5 that a ‘transition region’ with a certain thickness is formed between the water phase and the oil phase in the four systems. The surfactant molecules are mainly distributed in the ‘transition region’. A part of the surfactant molecules overlap with the water phase, which can reflect the interaction between polar groups and water molecules. The other part overlaps with the oil phase, reflecting the interaction between hydrophobic groups and the oil phase. A large number of sodium ions are concentrated in the range of the interaction between the polar group of the surfactant and water. This phenomenon is attributed to the electrostatic interaction between the sodium ions and the negatively charged sulfonate groups. In addition, the density distributions of the spacer groups show that the EG and NA groups are preferentially distributed toward the aqueous phase, whereas oil molecules are preferentially located near the CA and BR groups because of the hydrophobicity of these groups.

**Figure 6:**
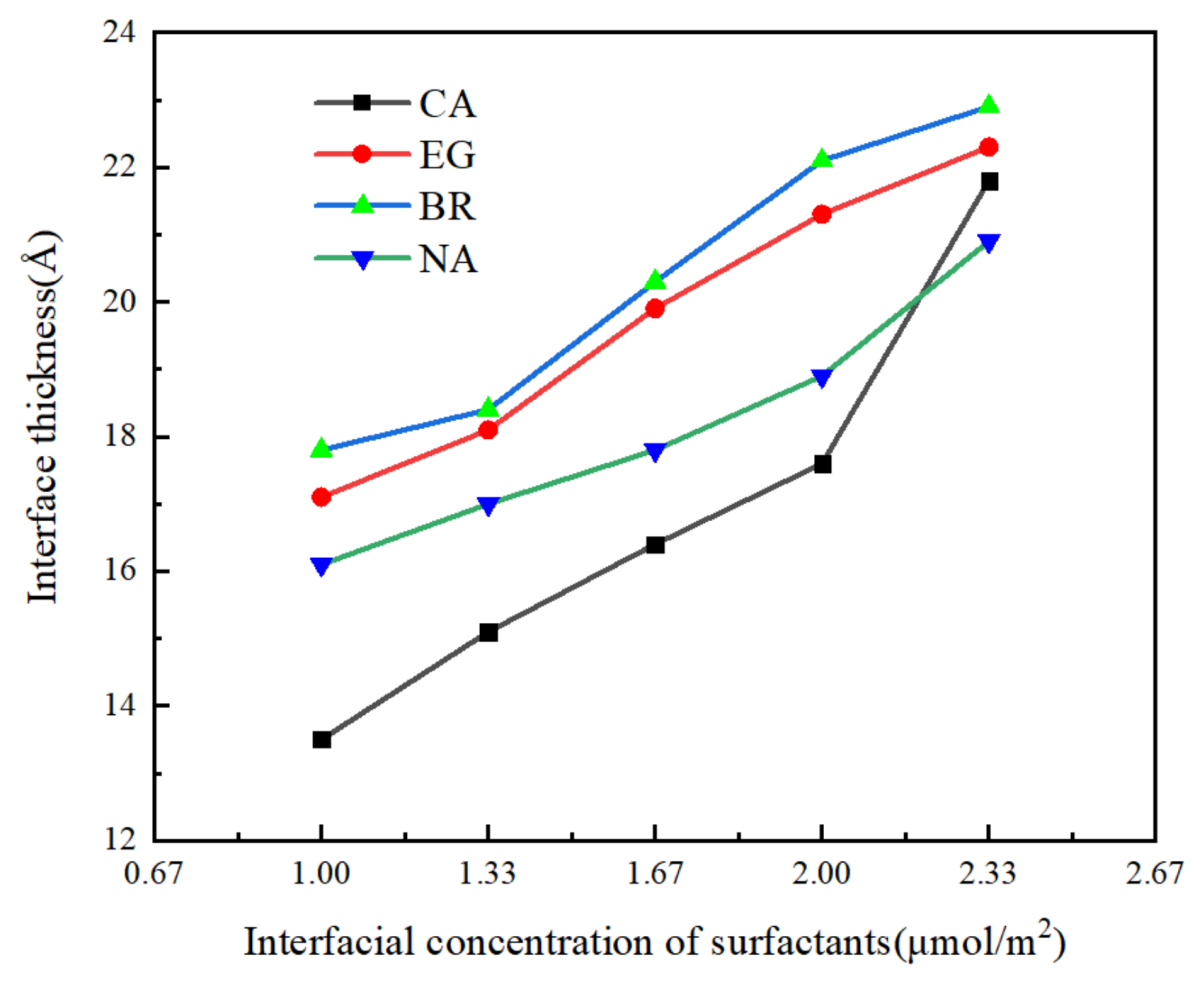
The interface thickness of each simulation system at different concentrations.

**Figure 7:**
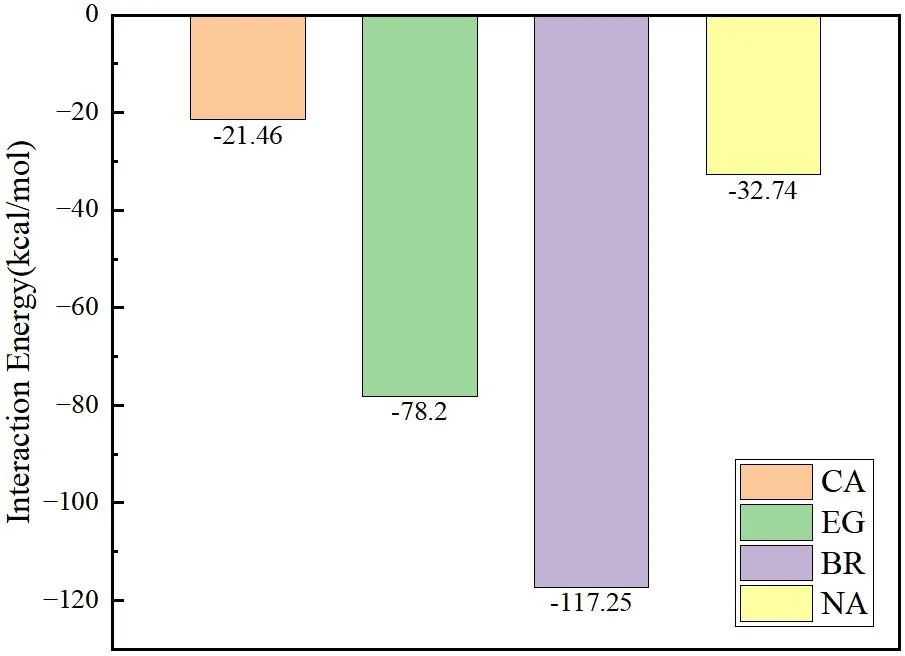
When the interfacial surfactant concentration is 2.00 μmol/m^2^, the interaction energy between different spacers and oil molecules.

Interfacial thickness, defined as the spatial extent of the boundary region between two immiscible phases, serves as an important parameter for characterizing the spatial extent of the oil–water transition region. In this study, interfacial thickness was calculated using the “90–90%” criterion, measuring the distance from the 90% density level of the bulk oil phase to that of the bulk water phase. This methodology represents a widely adopted standard in MD simulations, particularly for oil–water interfacial systems [69,70,71]. As shown in Figure 6, the interfacial thickness of all four systems gradually increased with rising surfactant concentration. Combined with the interfacial distributions observed in Figure 4, this trend can be attributed to the progressive accumulation and extension of surfactant adsorption layers at higher concentrations. At the same interfacial concentration, the BR system exhibited the largest interfacial thickness, followed by EG, whereas the NA and CA systems showed lower values. To clarify the role of the spacer groups, spacer–oil interaction energies were calculated. As shown in Figure 7, all values were negative, and the strengths of the attractive interactions followed the order BR > EG > NA > CA, which is consistent with the variation in interfacial thickness and suggests that stronger spacer–oil interactions promote the extension of interfacial layers.

Previous simulations of Gemini surfactants have shown that enhanced oil penetration into the interfacial region increases local oil–water mixing and broadens the interfacial transition region, resulting in a larger interfacial thickness [36]. A similar trend is observed for the BR system in the present study. Its rigid benzene-ring spacer partially extends into the oil phase and exhibits the strongest attraction to oil molecules, which may expand the distribution of interfacial components along the interfacial normal and thereby broaden the overall density transition region. Although the EG system does not exhibit the largest interfacial thickness, its ester-containing spacer enhances interfacial hydration and hydrogen-bond formation while maintaining moderate affinity toward the oil phase, thereby contributing to a more stable interfacial adsorption layer. Overall, interfacial thickness is governed by the combined effects of spacer structure, oil-phase affinity, and interfacial hydration behavior.

### 3.3. Interface Formation Energy and Interfacial Tension

**Figure 8:**
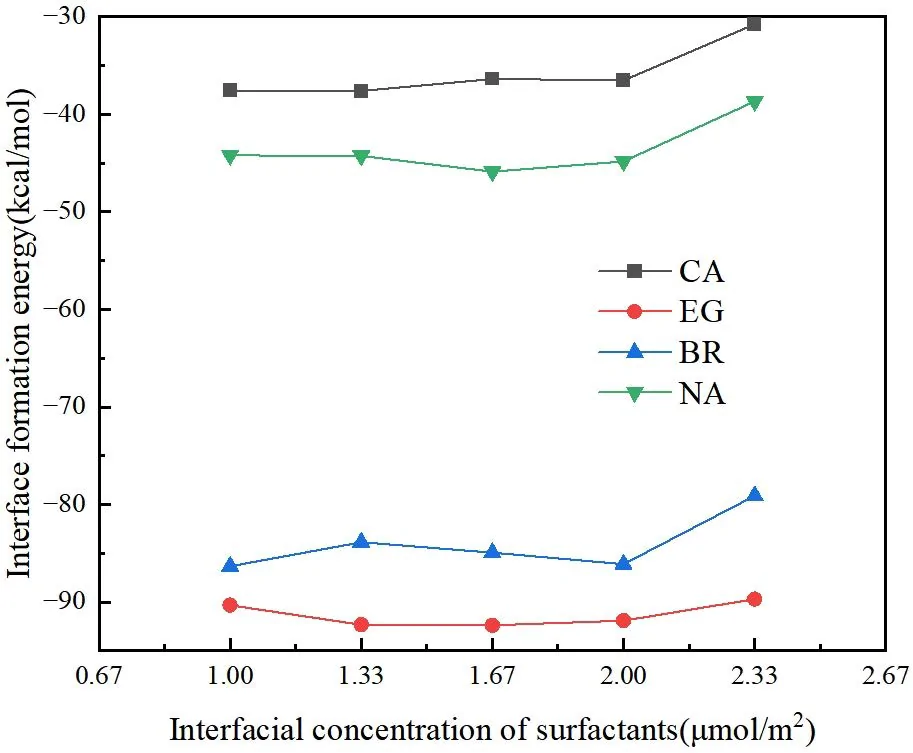
IFE of each system at different concentrations.

Interfacial formation energy (IFE) is an important parameter to evaluate the interfacial properties. It represents the decrease in energy upon the introduction of a surfactant molecule at the oil–water interface; therefore, IFE exhibits a negative value. IFE is calculated using Equation (4) [72]:

(4) $$\mathrm{IFE}=\frac{E_{\mathrm{total}}-(nE_{\mathrm{single}}+E_{\mathrm{oil}/\mathrm{water}})}{n}$$

where *E_total_* represents the total energy of the system, *E_single_* represents the energy of a single surfactant, *E_oil_*_/_*_water_* represents the total energy of the oil–water interface system without surfactants, and n represents the number of surfactants. As shown in Figure 8, the IFE values for all four surfactant systems are negative, indicating that each surfactant reduces the total energy of the oil–water interface, thereby lowering interfacial tension. Under identical simulation conditions, variations in IFE values reveal that differences in spacer groups influence the interfacial stability of gemini sulfonate surfactants. In general, the absolute value of the IFE followed the order EG > BR > NA > CA. This suggests that surfactants with ester-based spacers exhibited the strongest oil–water interface stabilization, followed by BR, NA, and CA. For each system, the IFE remained nearly constant over the interfacial surfactant concentration range of 1.00–2.00 μmol/m^2^. At 2.33 μmol/m^2^, however, its absolute magnitude decreased markedly, indicating less favorable interfacial stabilization. This reduction may be attributed to excessive surfactant loading at the interface, which induces interfacial curvature and promotes the transition toward microemulsion-like structures.

**Figure 9:**
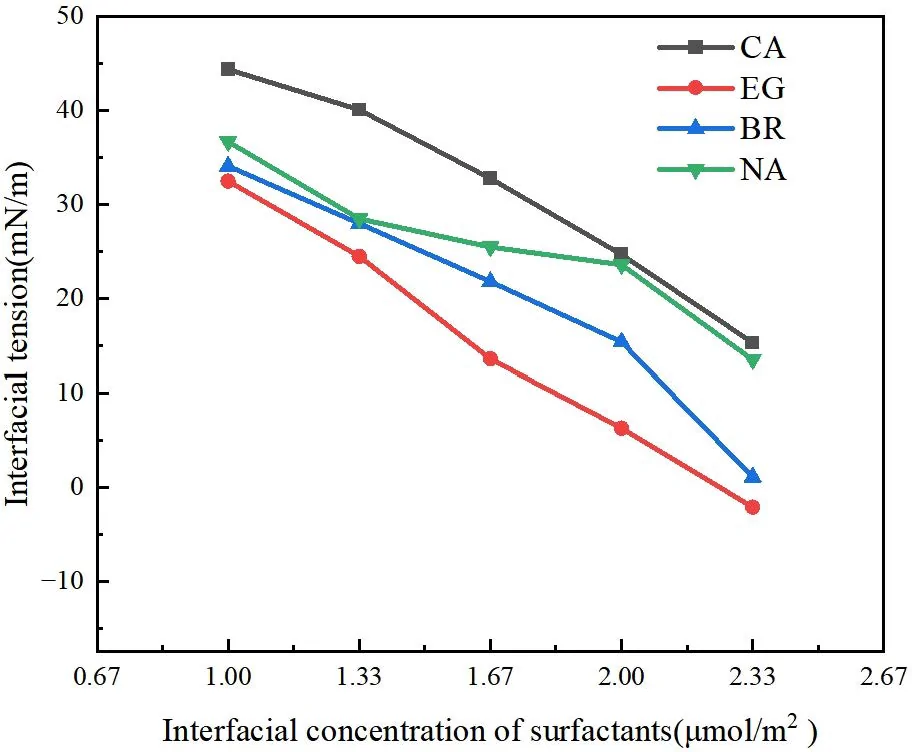
Interfacial tension of each system at different concentrations.

Subsequently, we calculated the interfacial tension of the four systems at different concentrations. The results are shown in Figure 9. A downward trend in interfacial tension was generally observed for the four systems as the surfactant concentration increased. At equivalent concentrations, the interfacial tension reduction efficacy followed the order: EG > BR > NA > CA, with the EG system demonstrating the strongest performance. Previous studies have shown that a decrease in oil–water interfacial tension is accompanied by an increase in the surface pressure of the interfacial adsorption film [71,73]. When the surface pressures on the two sides of the interfacial film become unequal, the resulting mechanical imbalance can cause the film to bend toward the side with higher surface pressure, thereby promoting the formation of microemulsion–like structures. Notably, at the maximum interfacial surfactant loading of 2.33 μmol/m^2^, the EG system yielded a negative value for its calculated interfacial tension. This nonphysical value arose because the pronounced interfacial curvature violated the planar-interface assumption underlying the pressure-tensor expression in Equation (3). Therefore, the calculated value at this concentration should not be interpreted as a physically meaningful interfacial tension. The observed transition from a planar adsorption layer toward microemulsion-like structures altered the interfacial geometry and rendered the planar–interface calculation method inapplicable.

### 3.4. Radial Distribution Function

**Figure 10:**
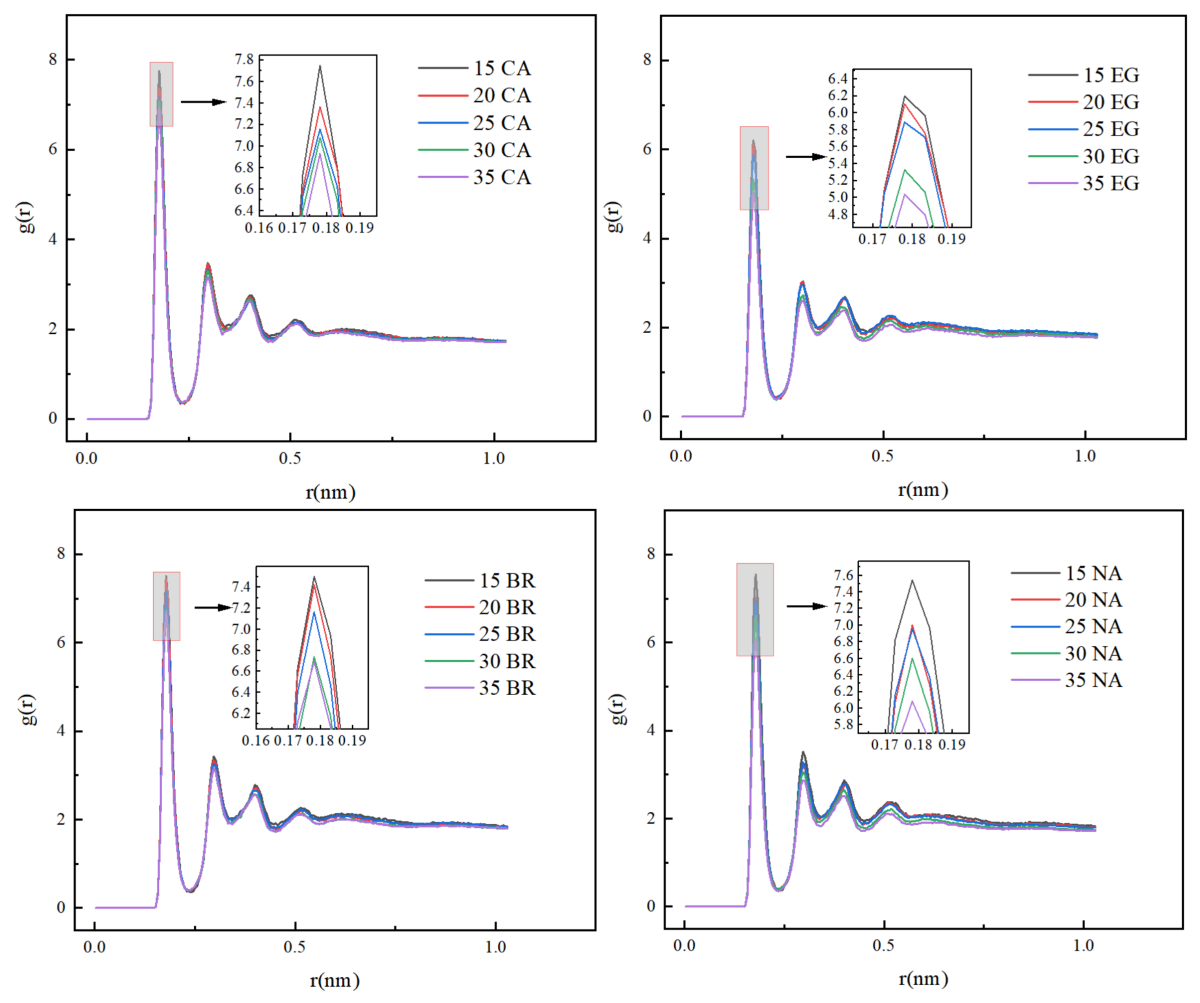
The radial distribution function of hydrophilic head group and water molecules in each system at different concentrations.

**Figure 11:**
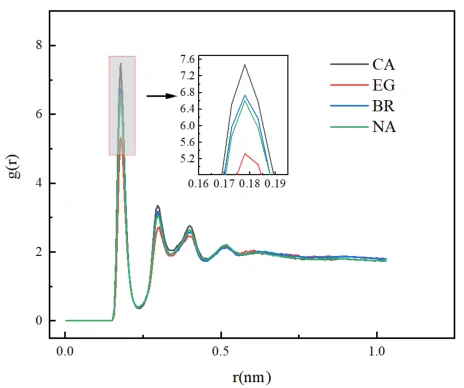
The radial distribution function of hydrophilic head group and water molecules for each system when the interfacial surfactant concentration is 2.00 μmol/m^2^.

To further elucidate the effects of spacer groups on interfacial hydration, the radial distribution functions (RDFs) between the hydrophilic headgroups and water molecules, as well as those between the spacer groups and water molecules, were calculated for all four systems. The RDF describes the relative probability of finding a particle or functional group at a given radial distance from a reference group and can therefore be used to characterize the spatial distribution of different components. The RDFs between the sulfonate headgroups and water molecules are shown in Figure 10. Four hydration shells were observed around the sulfonate groups in all systems. The first peak was located at approximately 0.18 nm, indicating that water molecules preferentially accumulated at this distance, thereby creating favorable spatial conditions for sulfonate–water hydrogen-bond formation. The peak positions remained nearly unchanged across the different systems and concentrations, indicating that neither the spacer structure nor the surfactant concentration markedly altered the characteristic interaction distance between the sulfonate headgroups and water molecules. The intensity of the first peak generally decreased with increasing surfactant concentration. At higher interfacial concentrations, increased steric crowding and reduced accessibility of water molecules to neighboring headgroups decreased the local accumulation of water molecules at the characteristic distance from the sulfonate groups. Figure 11 compares the sulfonate headgroup-water RDFs of the four systems at an interfacial concentration of 2.00 μmol/m^2^. The first-peak intensities followed the order CA > BR > NA > EG. Water molecules in the CA and BR systems exhibited stronger local accumulation at the characteristic distance from the sulfonate headgroups, resulting in higher firstpeak intensities. By contrast, the lower first peaks observed for EG and NA do not necessarily indicate weaker hydration, but rather reflect the redistribution of water molecules from the sulfonate-centered hydration shell toward the headgroup–spacer region.

**Figure 12:**
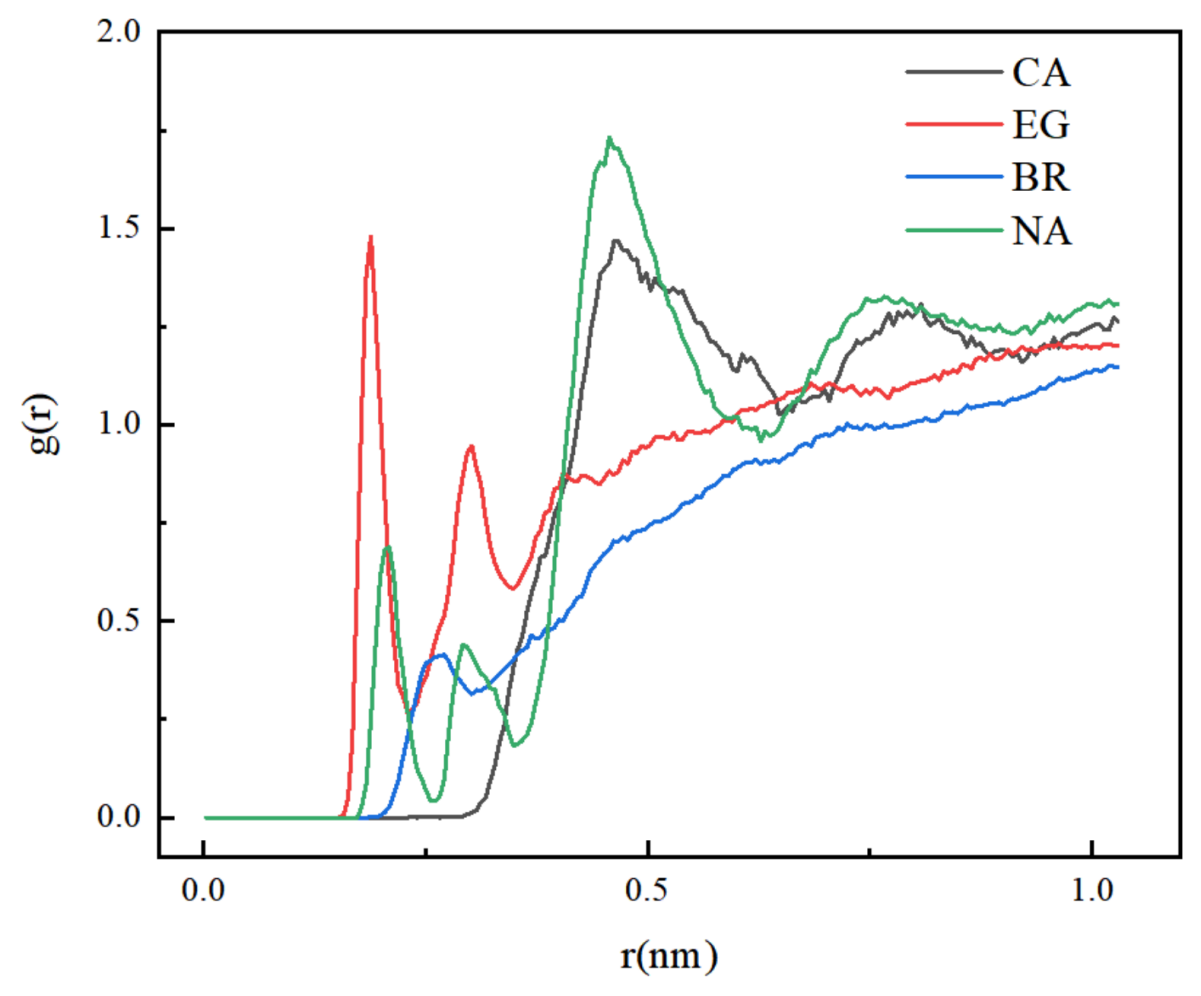
The radial distribution function of spacer group and water molecules for each system when the interfacial surfactant concentration is 2.00 μmol/m^2^.

The RDFs between the spacer groups and water molecules further revealed distinct hydration behaviors among the four systems. As shown in Figure 12, EG exhibited the highest first peak at approximately 0.19 nm, while NA showed a weaker peak at approximately 0.21 nm. In contrast, the characteristic peaks of BR and CA were located at approximately 0.27 and 0.46 nm, respectively, indicating weaker water enrichment around these spacer groups. The shorter spacer–water distances in EG and NA suggest that their polar spacers directly participate in interfacial hydration and redistribute water molecules within the headgroup–spacer region. By contrast, water molecules in BR and CA systems preferentially accumulated around the sulfonate headgroups rather than the spacer regions because of the hydrophobic nature of these spacers. It should be noted that the RDF reflects only local hydration, whereas the overall interfacial thickness (Figure 6) is mainly governed by the molecular structure of the spacer and its interaction with the oil phase. Therefore, despite the limited hydration contribution of the aromatic spacer itself, the BR system exhibits the largest interfacial thickness, highlighting the dominant role of spacer structure in regulating interfacial organization.

### 3.5. Orientational Distributions of Water Molecules

**Figure 13:**
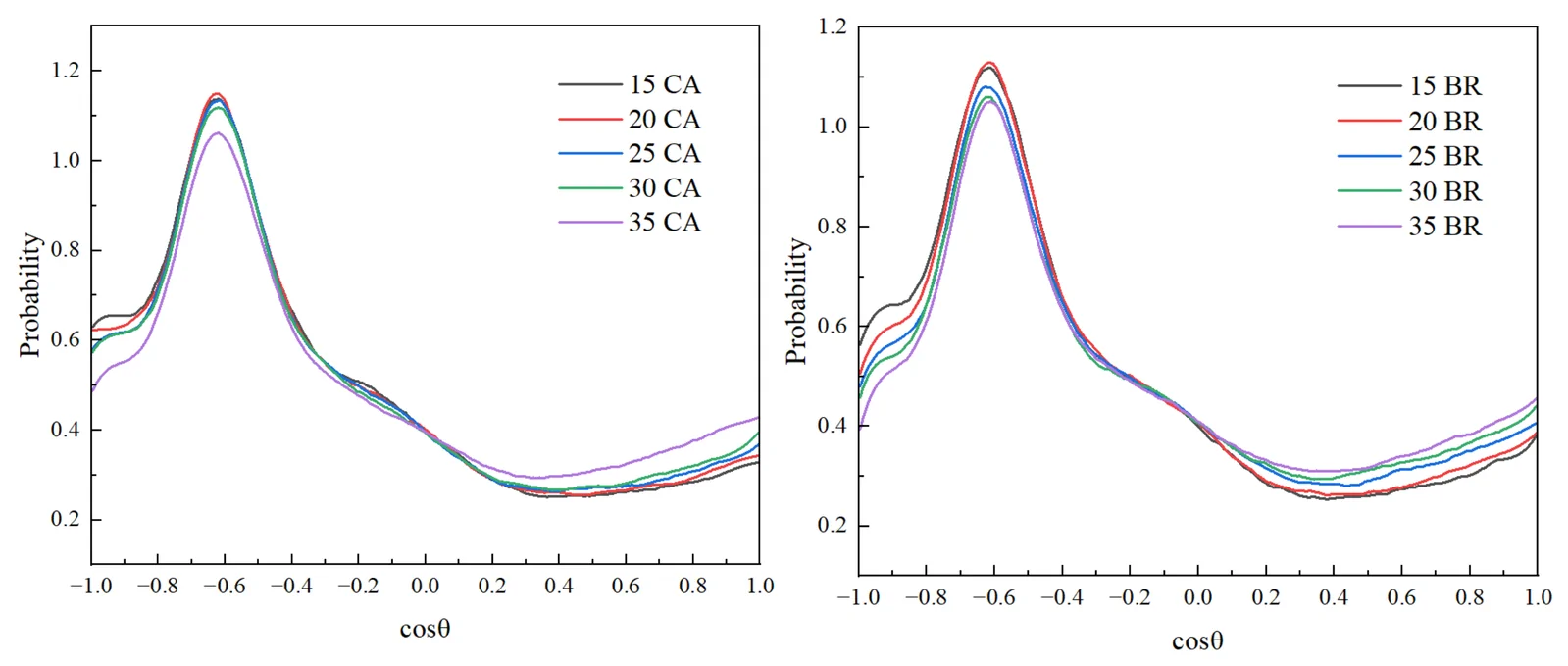

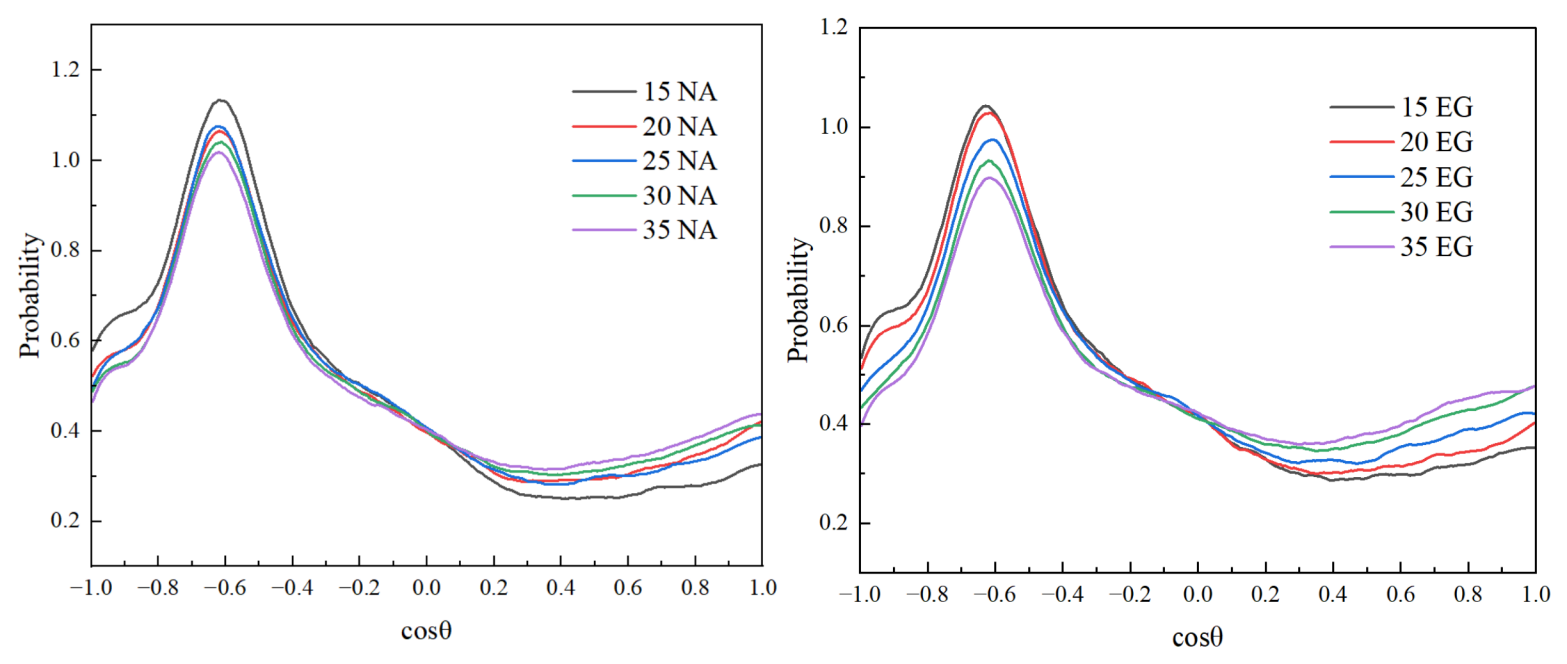
Orientational distributions of water around sulfonate oxygen atoms at different interfacial concentrations.

To further characterize the hydration structure around the sulfonate headgroups, the orientational distributions of water molecules within 5 Å of the sulfonate oxygen atoms were analyzed. The angle θ was defined as the angle between the vector from a sulfonate oxygen atom to a water oxygen atom and the vector from the water oxygen atom to the midpoint between its two hydrogen atoms. Accordingly, negative values of cosθ correspond to configurations in which the hydrogen side of the water molecule faces the sulfonate oxygen atom. As shown in Figure 13, all four systems exhibited a pronounced peak in the negative cosθ region, with the peak maxima located at cosθ values between −0.7 and −0.6. These peaks indicate that water molecules near the sulfonate oxygen atoms tended to orient with their hydrogen side toward the sulfonate oxygen atoms, providing favorable geometric conditions for sulfonate–water hydrogen bonding. The peak positions remained nearly unchanged across the different interfacial concentrations, indicating that the characteristic orientation of water molecules around the sulfonate groups was largely preserved.

With increasing interfacial concentration, the intensity of the peak in the negative cosθ region generally decreased, whereas the probability density at positive cosθ values generally increased. This trend indicates that the preferred orientation of water molecules with their hydrogen side facing the sulfonate oxygen atoms became less pronounced at higher interfacial concentrations. This change may be associated with the increasingly crowded interfacial environment at higher surfactant concentrations. Together with the RDF results, these findings indicate that increasing interfacial concentration affected both the radial and orientational distributions of water molecules around the sulfonate headgroups. The decrease in the first RDF peak reflects reduced local enrichment of water molecules at the characteristic distance, whereas the weakened peak in the negative cosθ region indicates reduced orientational ordering of water molecules around the sulfonate oxygen atoms.

### 3.6. Hydrogen Bonding

**Figure 14:**
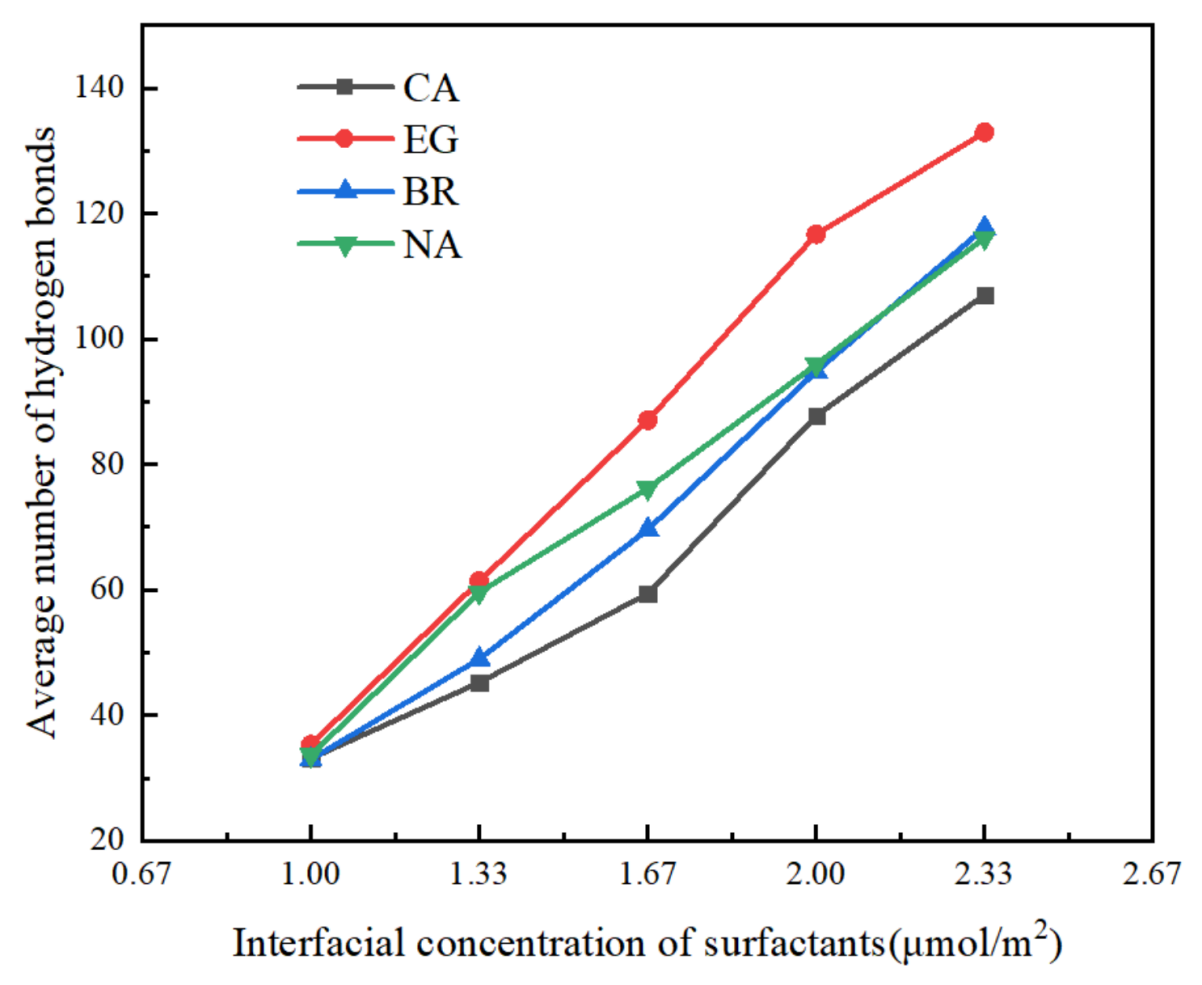
The average number of hydrogen bonds formed by hydrophilic head groups and water molecules in each system at different concentrations.

Hydrogen bonding between the sulfonate headgroups of the surfactants and water molecules contributes to the stabilization of the interfacial hydration structure. Accordingly, the average number of sulfonate–water hydrogen bonds was calculated for the four systems at different interfacial concentrations. As shown in Figure 14, the average number of hydrogen bonds increased progressively with increasing surfactant concentration in all four systems. This was primarily attributed to the increased number of sulfonate groups at the interface, which provided more available sites for hydrogen-bond formation. At the same interfacial concentration, the hydrogen–bond populations generally followed the order EG > NA > BR > CA, opposite to the trend observed for the sulfonate-water RDF peaks. This discrepancy indicates that hydrogen-bond formation is not solely determined by the local enrichment of water molecules, but also depends on their spatial distribution and orientational organization at the interface. In the EG and NA systems, the polar spacer groups promoted the redistribution of water molecules within the headgroup–spacer region, thereby reducing their local enrichment at the characteristic distance from the sulfonate oxygen atoms. Meanwhile, water molecules in the vicinity of the sulfonate groups retained a preferred orientation, with their hydrogen atoms directed toward the sulfonate oxygen atoms, thereby preserving geometrically favorable configurations for hydrogen-bond formation. Consequently, despite their relatively low sulfonate–water RDF peaks, EG and NA exhibited comparatively high hydrogen–bond populations. Recent MD studies using RDF and hydrogen–bond analyses have shown that oxygen–containing hydrophilic groups can enhance surfactant–water interactions and promote hydrogen–bond formation [74]. Although the RDF behavior reported in that study differs from the present system, the enhanced hydrogen–bonding tendency is consistent with the EG system, where the ester–containing spacer redistributes water within the headgroup–spacer region and gives the highest hydrogen–bond population among the four surfactants. These results indicate that interfacial hydration in gemini surfactant systems depends not only on the local enrichment of water molecules around the sulfonate headgroups but also on their spatial organization and orientational arrangement within the hydrophilic headgroup–spacer region.

### 3.7. Hydrophobic Tail-Chain Angle

**Figure 15:**
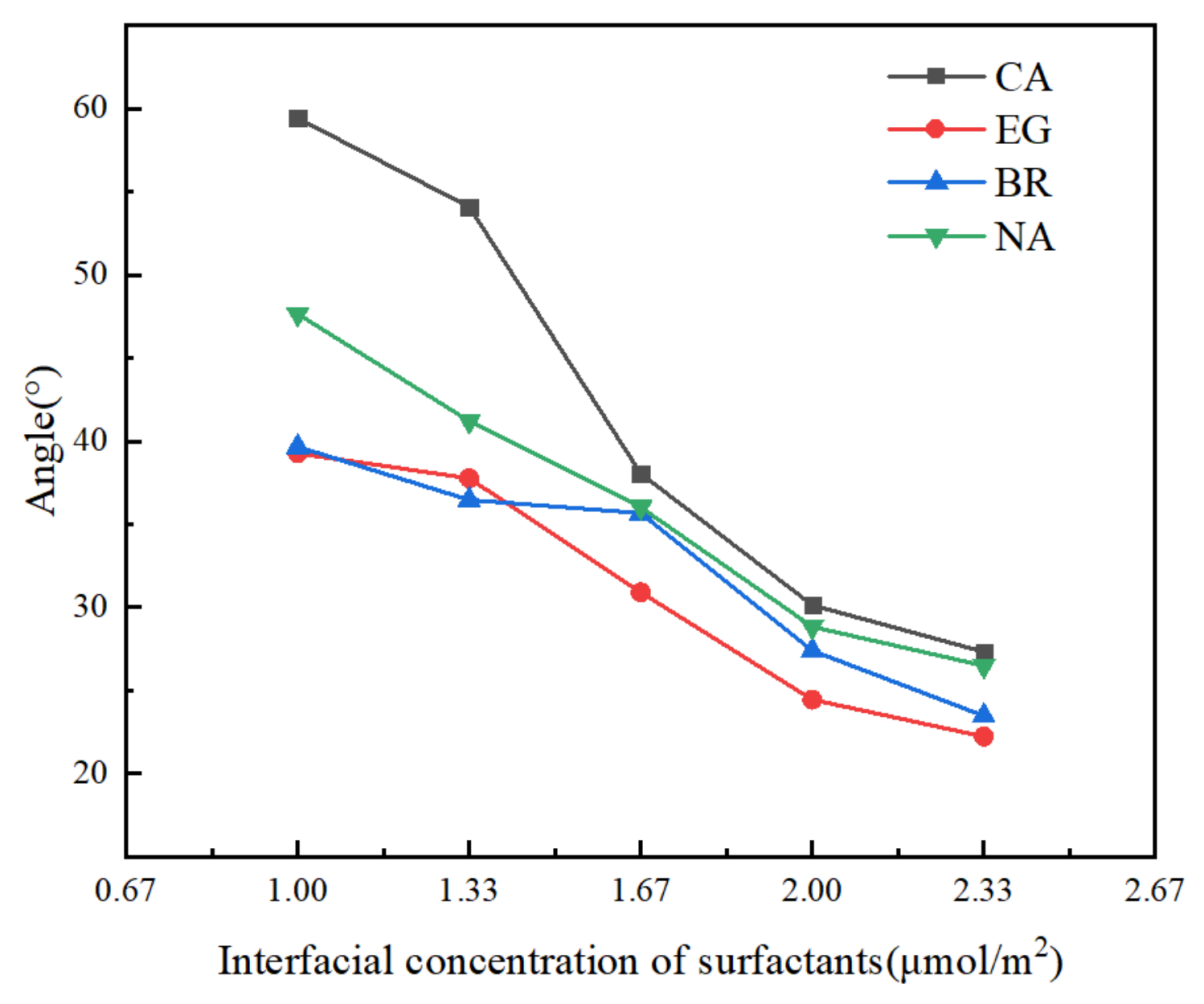
Hydrophobic tail-chain angle of surfactants in each system at different concentrations.

To clarify the influence of hydrophobic tail chains on the interfacial properties of different systems, the tail–chain angles of individual surfactant molecules were calculated. In this study, the hydrophobic tail–chain angle is defined as the angle between the vector connecting the first and last carbon atoms of the hydrophobic tail chain and the normal to the interfacial plane. A value closer to 90° indicates a tail-chain orientation parallel to the interface, while a value approaching 0° suggests a perpendicular alignment. Since the tail–chain angles of individual chains vary, all tail-chain angles from each frame during the last 1 ns were collected, and the angle with the highest occurrence probability was defined as the representative tail–chain angle. As shown in Figure 15, with increasing surfactant concentration, the tail–chain angles of all four surfactants decreased, indicating that the hydrophobic tail chains adopted a more perpendicular orientation relative to the interface. This phenomenon can be attributed to the structural reorganization of the interfacial adsorption layer. At high concentrations, the dense arrangement of surfactant molecules in the interfacial region reduces the lateral space available for individual molecules, forcing hydrophobic chains to adopt a more perpendicular orientation to alleviate steric hindrance while enhancing van der Waals interactions between neighboring chains, thereby forming a more ordered and compact interfacial structure. This concentration–dependent reorientation is consistent with recent atomistic simulations showing that increasing surfactant surface coverage leads to a more upright molecular orientation at the interface [75], in agreement with the decrease in tail–chain angle observed in the present study. It is worth noting that Figure 15 further reveals distinct structure–property correlations in tail–chain orientation at identical concentrations, with the angle order following CA > NA > BR > EG. This highlights the key influence of spacer chemistry on molecular configuration. For the EG system, the presence of an ester spacer was associated with the smallest tail–chain angle, indicating that hydrophobic tails were more inclined toward a perpendicular orientation. This orientation promotes denser interfacial molecular packing, which contributes to the reduction in oil–water interfacial tension.

### 3.8. Effects of PAM and HPAM on the EG System

Based on the above results, the EG system exhibited favorable interfacial performance among the four gemini surfactants. Therefore, the EG system at an interfacial concentration of 1.67 μmol/m^2^ was selected to further investigate the effects of PAM and HPAM on its interfacial behavior. The effects of the polymers were evaluated by comparing the density distributions and interfacial tensions of the EG, EG–PAM, and EG–HPAM systems, together with the interaction energies between the polymers and the surfactant headgroups, spacer groups, and water molecules. To assess the stability of the simulated systems, we evaluated the time evolution of the total energy and polymer–surfactant interaction energies. As shown in Figure S1, the total energy fluctuated around a relatively stable average value, while Figure S2 shows that the interaction energies remained within a relatively stable range during the final 2 ns of the production run.

**Figure 16:**
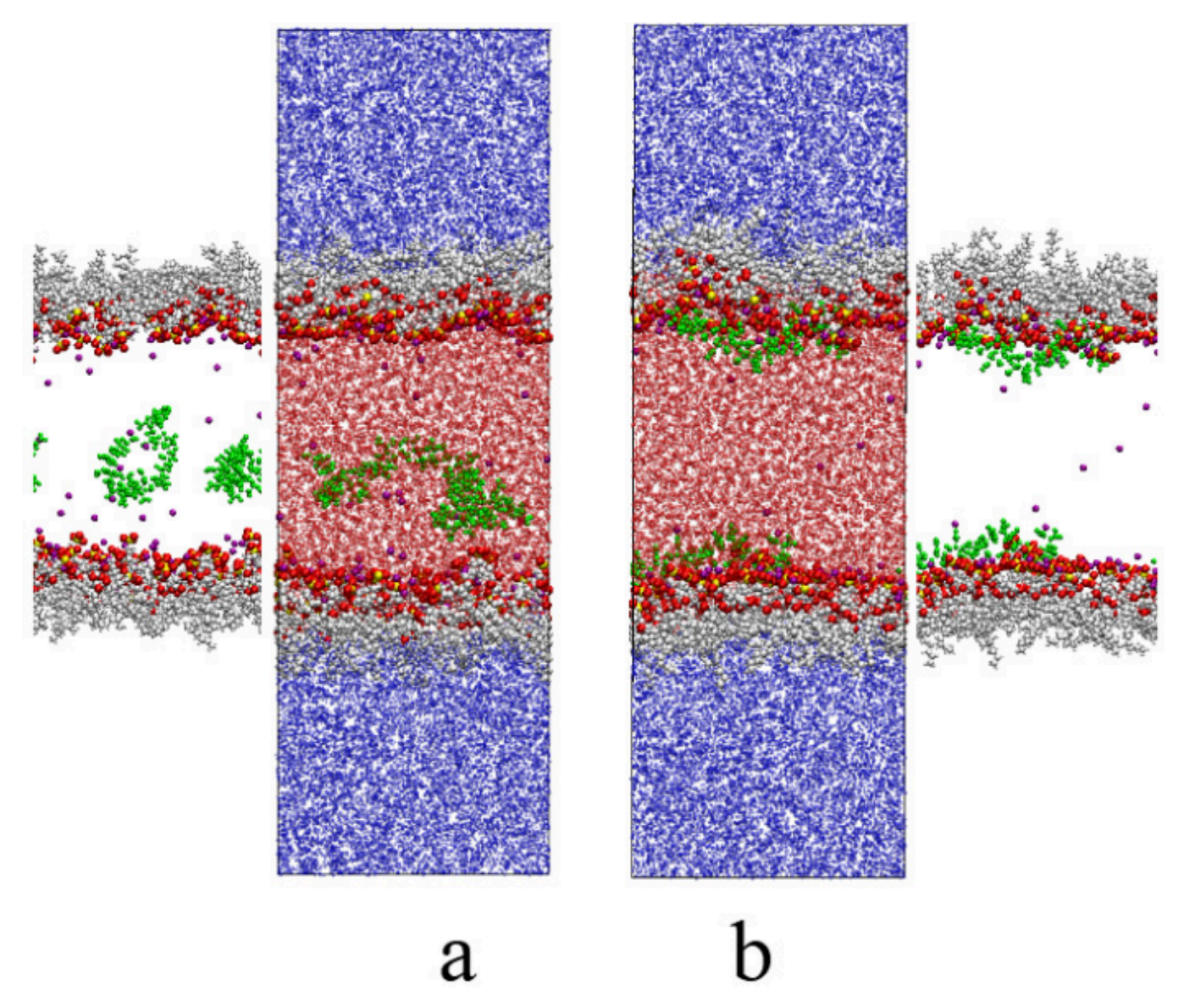
The last frame snapshots after the simulation of the EG–HPAM and EG–PAM systems, together with the corresponding front and top views after removing the water and oil phases. The polymer chains are shown in green. (**a**) EG–HPAM; (**b**) EG–PAM.

**Figure 17:**
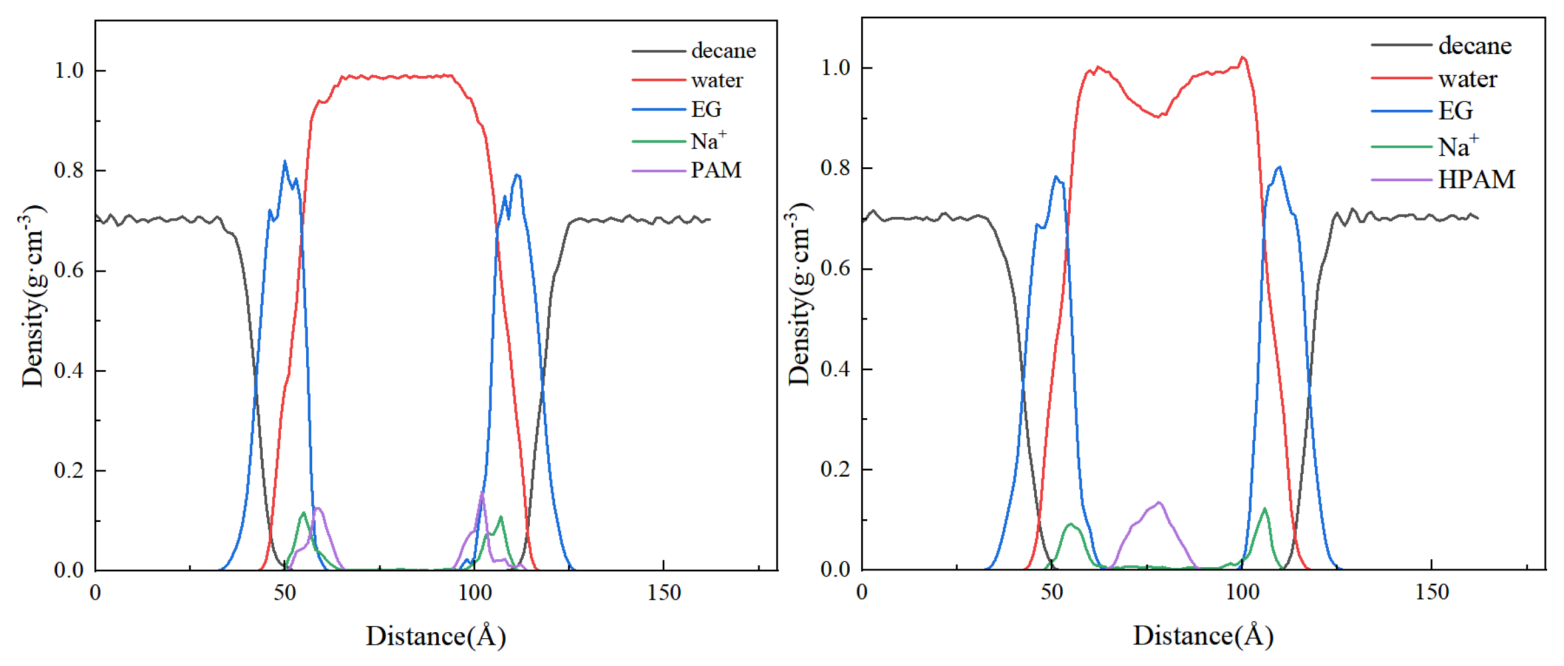
Density profiles of each component along the z–axis at an interfacial surfactant concentration of 1.67 μmol/m^2^.

Figure 16 shows that HPAM is mainly distributed in the aqueous phase and is clearly separated from the EG interfacial layer, whereas PAM is located near the oil–water interface and remains in contact with EG. The density distributions further confirm this difference. As shown in Figure 17, PAM exhibits distinct density peaks at approximately 55 and 110 Å, corresponding closely to the EG interfacial regions. In contrast, the HPAM density is concentrated in the aqueous region between the two EG interfacial peaks, with its main peak located within the bulk water phase. These results indicate that PAM preferentially accumulates near the EG adsorption layer, whereas HPAM predominantly resides in the bulk aqueous phase. Wu et al. [52] similarly showed that PAM–SDS interactions can affect the distribution and hydration characteristics of the surfactant interfacial layer. Previous studies [76] have also shown that the hydrolysis degree of HPAM significantly affects its distribution and aggregation behavior at oil–water interfaces.

**Figure 18:**
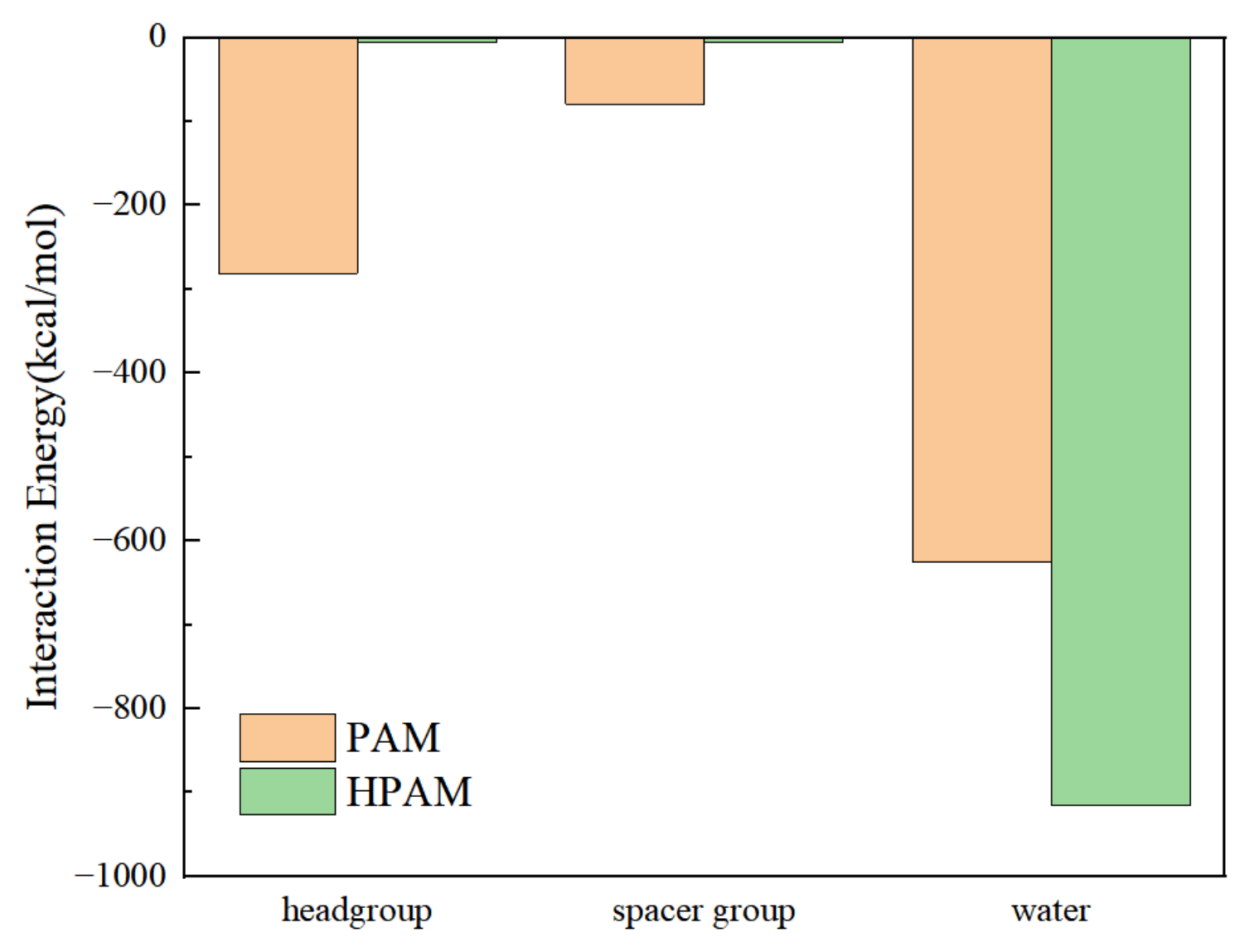
Average interaction energies of PAM and HPAM with the headgroups and spacer groups of the EG gemini sulfonate surfactant, as well as with water molecules.

To further explain the different spatial distributions of PAM and HPAM, the interaction energies between the polymers and the EG headgroups, spacer groups, and water molecules were calculated. As shown in Figure 18, PAM exhibits strong attractive interactions with the EG headgroups and spacer groups, resulting in its stable enrichment near the interface. After hydrolysis, the interactions of HPAM with EG are greatly weakened, whereas its interactions with water are markedly enhanced. The charged groups introduced by hydrolysis increase the affinity of HPAM for the aqueous phase, making polymer–water interactions dominant and favoring its distribution in the bulk water phase rather than near the EG adsorption layer. This result is consistent with the molecular snapshots and density distributions.

Table 2 shows that both PAM and HPAM reduce the interfacial tension of the EG system, but to markedly different extents. The addition of PAM decreases the interfacial tension from approximately 13.5 to 2.089 mN/m, whereas HPAM only reduces it to 13.05 mN/m. Combined with the molecular snapshots, density distributions, and interaction-energy results, PAM is enriched near the oil–water interface and exhibits strong attractive interactions with the EG headgroups and spacer groups. This close association with the EG interfacial layer is consistent with the pronounced reduction in interfacial tension. Previous studies have likewise reported synergistic interfacial effects between polyacrylamide and surfactants at oil–water interfaces, indicating that polymer–surfactant association can substantially modify interfacial properties [77]. In contrast, HPAM interacts predominantly with water and is mainly distributed in the bulk aqueous phase, while its attraction toward EG is greatly weakened. Consequently, its influence on the EG interfacial layer is limited, corresponding to only a slight change in interfacial tension. Studies of HPAM-gemini surfactant systems at the decane–water interface have also shown that HPAM can alter the structure and interfacial response of mixed adsorption layers [78]. Therefore, the different interfacial-tension responses of PAM and HPAM are closely related to their interfacial distributions and the strength of their interactions with EG.

Owing to the limitations of the simulation box size and system configuration, two oligomer chains with a degree of polymerization (DP) of 20 were used to represent PAM and HPAM. The effects of chain length, concentration, and degree of hydrolysis were not systematically investigated. Increasing polymer chain length and concentration can enhance chain entanglement and steric hindrance, restrict chain mobility, and prolong conformational relaxation, thereby affecting the interfacial film structure. In addition, charged groups introduced through hydrolysis can enhance electrostatic repulsion between monomers, leading to more extended HPAM chain conformations and increasing solution viscosity under appropriate degrees of hydrolysis and solution conditions. These factors may affect polymer adsorption at the interface and its diffusion between the interfacial region and the bulk aqueous phase, although their specific effects require further investigation. Therefore, the present results reflect the effects of oligomers on oil–water interfacial properties.

## 4. Conclusions

In this study, the effects of four gemini sulfonate surfactants (BR, EG, NA, and CA) with distinct spacer groups on oil–water interfacial properties were systematically investigated using all-atom MD simulations. The main conclusions are as follows:

(1) Interfacial thickness is governed by the combined effects of spacer structure, oil-phase affinity, and interfacial hydration. The rigid BR spacer, with its strong affinity toward the oil phase, broadens the interfacial transition region, whereas the polar EG spacer promotes interfacial hydration and hydrogen-bond formation, thereby stabilizing the interfacial adsorption layer.
(2) Within the concentration range where the interface remains approximately planar, all four surfactants effectively reduce oil–water interfacial tension, with interfacial activity following the order EG > BR > NA > CA. The absolute IFE magnitudes follow the same order, supporting the superior interfacial stabilization capability of the ester-containing EG spacer. At 2.33 μmol/m^2^, excessive interfacial loading induces pronounced curvature and a transition toward microemulsion-like structures, rendering the planar-interface tension calculation inapplicable and decreasing the absolute IFE magnitude.
(3) Spacer polarity and molecular configuration jointly regulate the interfacial hydration network and packing organization. Polar spacers facilitate effective hydrogen bonding within the headgroup–spacer region, while spacer-dependent tail-chain orientations determine packing compactness. Among the four systems, EG achieves the most favorable combination of hydration and molecular organization, contributing to its superior interfacial performance.
(4) PAM and HPAM exert different effects on the EG system through distinct interaction pathways. PAM strongly interacts with the EG headgroups and spacer groups and accumulates at the interface, leading to a pronounced reduction in interfacial tension. In contrast, hydrolysis strengthens HPAM–water interactions and promotes the preferential distribution of HPAM in the bulk aqueous phase, thereby weakening its influence on the EG interfacial layer.

However, this work employed a simplified n-decane/water model and therefore did not account for the effects of oil-phase composition and aqueous-phase salinity on interfacial organization. Future work will extend the model to brine systems and more representative oil phases containing aromatic hydrocarbons or asphaltene-like components to further investigate their effects on spacer-dependent adsorption, molecular packing, and interfacial stabilization.

## Figures and Tables

**Table 1:** Compositions of the simulation systems.

System	Decane	Surfactant	H_2_O	Polymer	Na^+^
1	700	-	5000	-	-
2	700	30	5000	-	60
3	700	40	5000	-	80
4	700	50	5000	-	100
5	700	60	5000	-	120
6	700	70	5000	-	140
EG-PAM	700	50	5000	2 PAM	100
EG-HPAM	700	50	5000	2 HPAM (20%)	108

Note: Four surfactant types, namely CA, EG, NA, and BR, were investigated at five different interfacial concentrations. The EG-PAM and EG-HPAM systems were constructed based on the EG system at 1.67 μmol/m^2^.

**Table 2:** Interfacial tensions of the EG, EG-PAM, and EG-HPAM systems.

System	Interfacial Tension (mN/m)
EG	13.5
EG-PAM	2.089
EG-HPAM	13.05

## Data Availability

The original contributions presented in this study are included in the article. Further inquiries can be directed to the corresponding author.

## Supplementary Materials

The following supporting information can be downloaded at: https://www.mdpi.com/article/10.3390/polym18212665/s1, Figure S1: Time evolution of the total energy of PAM (a) and HPAM (b) systems, Figure S2: Time evolution of polymer-surfactant interaction energies with the surfactant headgroups and spacer groups: (a) PAM system; (b) HPAM system.
