# Supplementary material for: Molecular Insights into Oil–Water Interfacial Behavior of Gemini Sulfonate Surfactants: The Critical Role of Spacer Group
Source: Polymers (Basel). 2026 Oct 28;18(21):2665. doi: 10.3390/polym18212665 (PMC13765601; doi:10.3390/polym18212665)
Supplement: Supplementary file 1 [file polymers-18-02665-s001.zip › polymers-4646786-supplementary.pdf]

Supplementary Material

Molecular Insights into Oil-Water Interfacial Behavior of Gemini Sulfonate Surfactants: The Critical Role of Spacer Group

WenRen [†,ab], *Hao Lu* [†,c], *Jinyuan Zhang* [c], *Xingchun Li* [b] , *Linglan Zhang* [c], *Chunmao Chen* [a],*Qinghong Wang* [a], *Shuixiang Xie* [b], *Ling Lin* [c,*]

[a] College of Chemical Engineering and Environment, China University of Petroleum-Beijing, State Key Laboratory of Heavy Oil Processing, Beijing 102200, China

[b] CNPC Research Institute of Safety and Environment Technology, State Key Laboratory of Petroleum Pollution Control, Beijing 102206, China

[c] School of Chemistry and Chemical Engineering, Southwest Petroleum University, Chengdu 610500, Sichuan, China.

[†] These authors contributed equally to this work.

Corresponding Author

Ling Lin – School of Chemistry and Chemical Engineering, Southwest Petroleum University; Phone: +86 28 83037306; Email: cowbolinling@aliyun.com

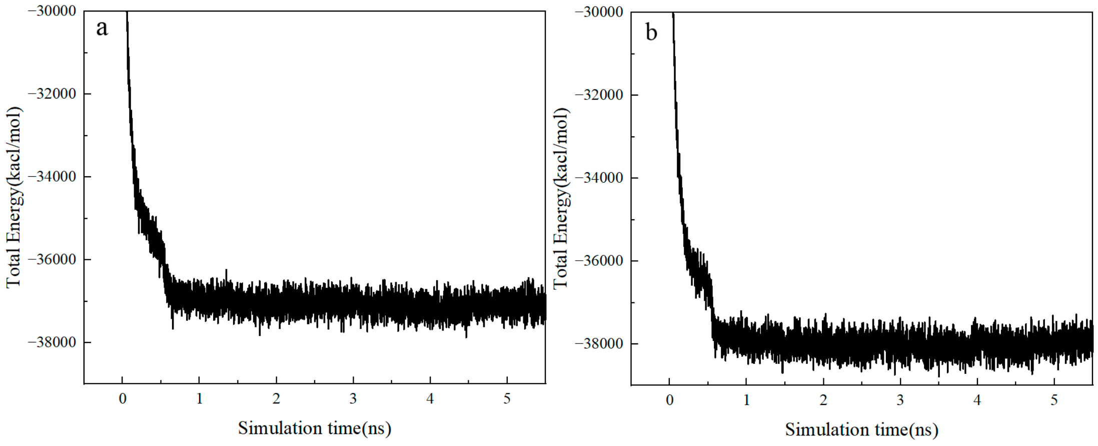

Figure S1.Time evolution of the total energy of PAM (a) and HPAM (b) systems.

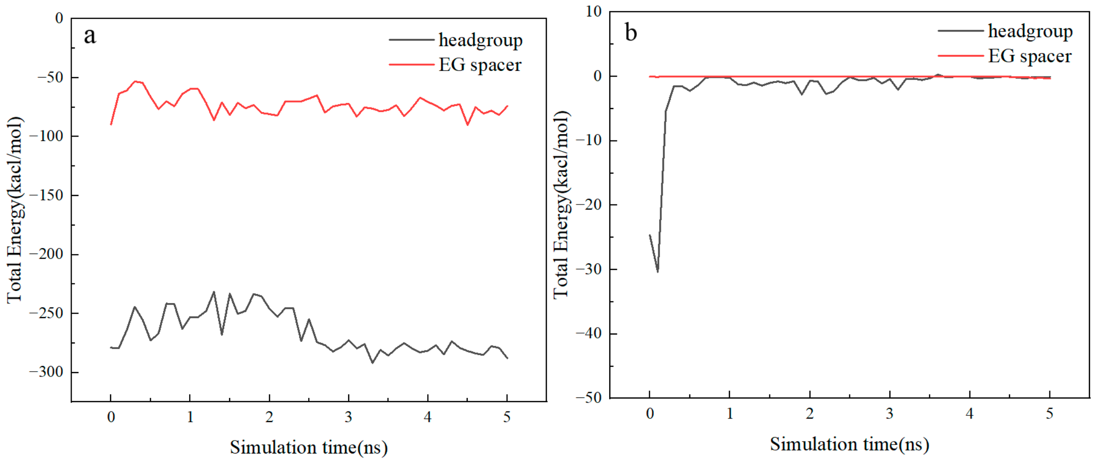

Figure S2. Time evolution of polymer-surfactant interaction energies with the surfactant headgroups and spacer groups: (a) PAM system; (b) HPAM system.
